# Congenital Peritoneopericardial Diaphragmatic Hernia in a Dog: Ventral Advancement Diaphragmatic Technique for Repair

**DOI:** 10.1155/crve/9977947

**Published:** 2026-04-17

**Authors:** Jonas D. Perez-Bello

**Affiliations:** ^1^ Institute of Experimental Surgery, Faculty of Medicine, Universidad Central de Venezuela, Caracas, Venezuela, ucv.ve

**Keywords:** congenital, diaphragm, hernia, peritoneopericardial, surgery, ventral advancement

## Abstract

Peritoneopericardial diaphragmatic hernia (PPDH) is a rare congenital anomaly that requires surgical treatment. Its clinical presentation varies and can affect one or more abdominal organs. This case report refers to a recently described surgical technique called ventral advancement of the diaphragm (VADT). Previously, a complete diagnostic approach was made in a 4‐month‐old, 6 kg (13.22 lbs.), uncastrated, mixed breed dog, with a history of cough from the time of adoption, with no known record of trauma. Physical examination revealed tachycardia, tachypnea, body condition score of 5/9, positivity to tracheal sensitivity tests, and attenuation of heart sounds in the left hemithorax, with no other relevant alterations. A PPDH was diagnosed and surgical correction was recommended. VADT was performed by incising the diaphragm ventrally with scissors, following the costal arch, about 3 cm on each side, avoiding excessive tension on the wound and possible postoperative dehiscence. Nonabsorbable monofilament material was used, with an interrupted horizontal suture pattern, without intraoperative or postoperative complications. The results showed that VADT is an easy surgical procedure to perform, being its approach beneficial for the patient, when compared with other techniques, providing an effective clinical resolution; generating little tension on the sutures that correct the diaphragmatic defect, with low rate of complications, dehiscence or recurrences, as reported in the literature and observed in this case. It is concluded that VADT is a surgical technique that is not very demanding for the surgical team and allows the defect to be corrected effectively, confirming its efficacy; however, further clinical evidence is recommended to validate its reproducibility.

## 1. Introduction

Peritoneopericardial diaphragmatic hernia (PPDH) is a rare form of congenital hernia and occurs when there is a communication between the abdomen and the pericardial sac. Synonymous terms include the following: pericardial diaphragmatic hernia and congenital hernia [1–4]. This malformation allows the passage of abdominal contents such as the liver, gallbladder, spleen, stomach, bowel loops, or omentum through the defect into the pericardial sac and can result in dyspnea, collapse, and death from pulmonary compression and visceral entrapment, causing respiratory, cardiac, or gastrointestinal clinical signs [1, 3, 4]. It occurs due to abnormal formation or fusion of the transverse septum during embryologic development, leading to incomplete separation of the pericardial and peritoneal cavities. It can be caused by teratogenic agents, genetic defects, or prenatal injury [1, 2]. Its prevalence has been reported in 0.015% of dogs and is usually accompanied by other congenital abnormalities, such as umbilical hernias, abdominal wall hernias, or sternal anomalies, in 57.1% of cases [4].

**Figure 1:**
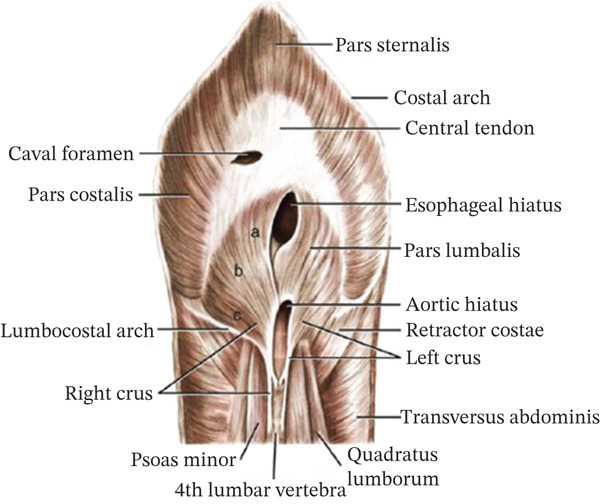
Abdominal surface of the diaphragm (Source: Hermanson et al. [5]).

The diaphragm is a muscle‐tendinous membrane about 5–6 mm thick, which separates the thoracic cavity from the abdominal cavity. It consists of a central tendinous portion, surrounded by a muscular portion and through it pass the caudal vena cava, esophagus, aortic artery, azygos and hemiazygos veins, thoracic duct and vagus nerves (Figure 1) [1, 5]. Clinical signs of PPDH may include tachypnea, dyspnea, cough, anorexia, depression, vomiting, diarrhea, thinning, wheezing, exercise intolerance, pain after eating, or all of the above. The most frequently incarcerated organ is the liver and associated pericardial effusion is frequently observed [2, 4, 6]. Physical examination may reveal a heart murmur, attenuation of cardiac and respiratory sounds, and, in addition, borborygmus within the thoracic cavity [4].The severity and number of clinical signs are usually correlated with the size of the defect, as small defects may be occluded by fatty sickle cell ligament, resulting in symptomless pictures [4].

Radiology is routinely used for diagnosis. For this purpose, ventro‐dorsal and lateral images of the thorax are performed, in which cardiomegaly, increased cardiac silhouette, presence of tubular organs with gas within the pericardial sac, poor definition or discontinuity of the diaphragmatic borders, and loss of distinction between the heart and the diaphragm may be evident [4, 6]. Echocardiography may not allow a complete evaluation of the heart due to anomalous position or compression of the heart, but ventricular septal defects, mitral regurgitation, tricuspid dysplasia, or subaortic stenosis may be noted [4]. Abdominal ultrasound is also very useful for diagnosis, demonstrating disruption of the diaphragm, cranial hepatic asymmetry, and the presence of abdominal viscera within the thoracic cavity [7]. The treatment of choice for this type of hernia is diaphragmatic herniorrhaphy [4]. The ideal age for repair is between 8 and 16 weeks, since adhesions are unlikely to occur and the laxity of the surrounding tissues facilitates defect closure without tension [2].

The surgery is carried out through a ventral midline abdominal incision, with the patient placed in the inverted Trendelenburg position, which facilitates the reduction of the herniated contents. Any adhesions should be dissected and, if necessary, the defect enlarged [1, 2, 6]. Recently, a technique for closure of these defects, called the ventral advancement diaphragmatic technique (VADT), has been described and the use of autologous flaps and surgical mesh has also been reported [3]. VADT is advantageous since it does not pose great technical difficulty and has a lower risk of complications for the patient.

The procedure consists of ventral advancement of the defect following the costal arch, allowing closure of the anomaly, without generating tension on the sutures. The closure of the defect can be done with absorbable or nonabsorbable sutures, both in continuous and discontinuous pattern, by making apposition of the diaphragmatic remnants, without closing the pericardial sac [1, 2, 4, 6]. The air present in the pleural cavity must be removed, as well as the fluid drained, by means of a thoracotomy tube or pleural probe, which is maintained in the immediate postsurgical period, being removed 12 to 24 h after surgery, when the drainage volume is less than 2.2 mL/kg and the negative pressure of the thorax has been reached [1, 2, 4, 6]. Intraoperative complications are not usually reported in dogs; the prognosis is good after surgery, and 85% of patients have improved clinical signs [2, 4]. Postoperative complications have an acceptable incidence and are usually associated with self‐trauma or surgical wound infection [6].

## 2. Case Report

A 4‐month‐old, unneutered, 6 kg male mixed breed dog, with a history of coughing since adoption, 30 days earlier and no known history of trauma, was brought to the clinic for medical evaluation. Physical examination revealed the presence of tachycardia (180 bpm), tachypnea (80 rpm), body condition score of 5/9, positivity to tracheal sensitivity tests, and attenuation of heart sounds in the left hemithorax. No other relevant alterations were detected.

### 2.1. Diagnostic Approach

**Figure 2:**
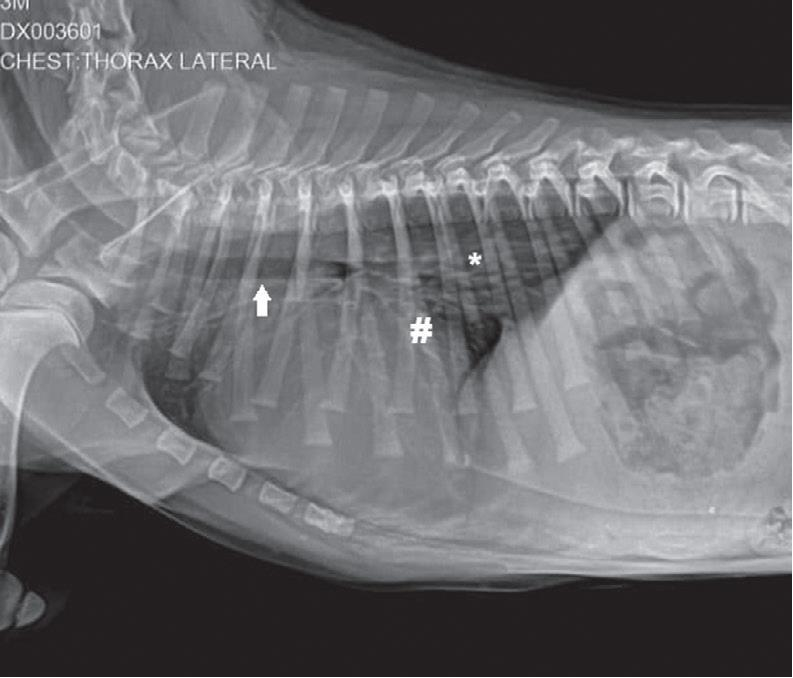
LLRL chest radiograph. Dorsal displacement of the trachea (∗), globular cardiac silhouette (#), interstitial and bronchial pattern (arrow) in caudal pulmonary lobes, suggestive of generalized cardiomegaly and bronchopneumonia.

**Figure 3:**
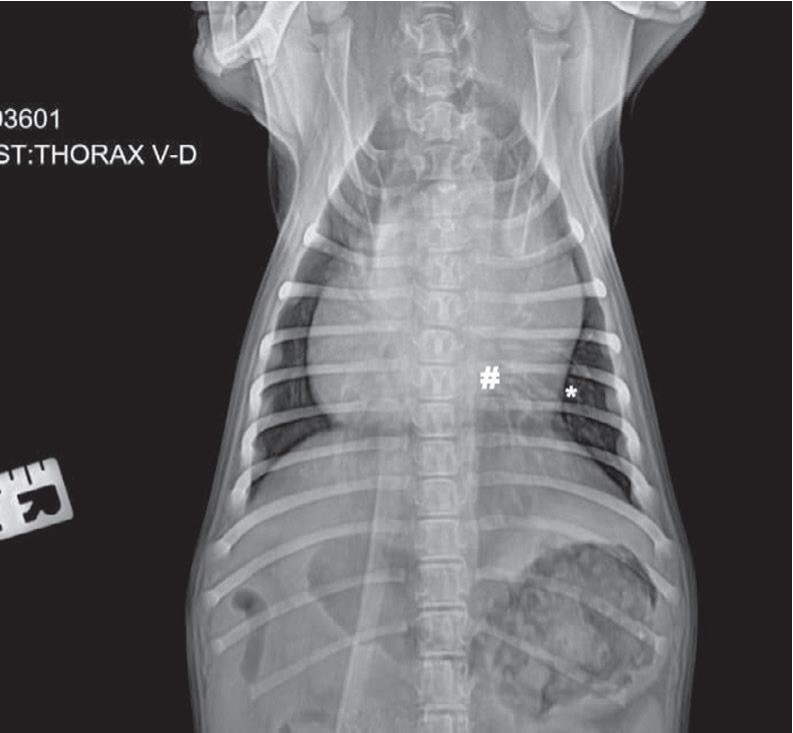
DV chest radiograph. Globular cardiac silhouette (#), interstitial and bronchial pattern (∗), suggestive of generalized cardiomegaly and bronchopneumonia.

A general diagnostic panel was conducted, including a complete blood count, blood biochemistry, left lateral, right lateral (LLRL) and dorso‐ventral (DV) chest radiographs. Hematology and biochemical profile results were within normal ranges, without significant alterations. The radiographic study showed a dorsal displacement of the trachea, a globular cardiac silhouette, and an interstitial and bronchial pattern in the caudal pulmonary lobes, suggesting generalized cardiomegaly and bronchopneumonia (Figures 2 and 3); however, this bronchial pattern was considered secondary to the compression of the hernia.

**Figure 4:**
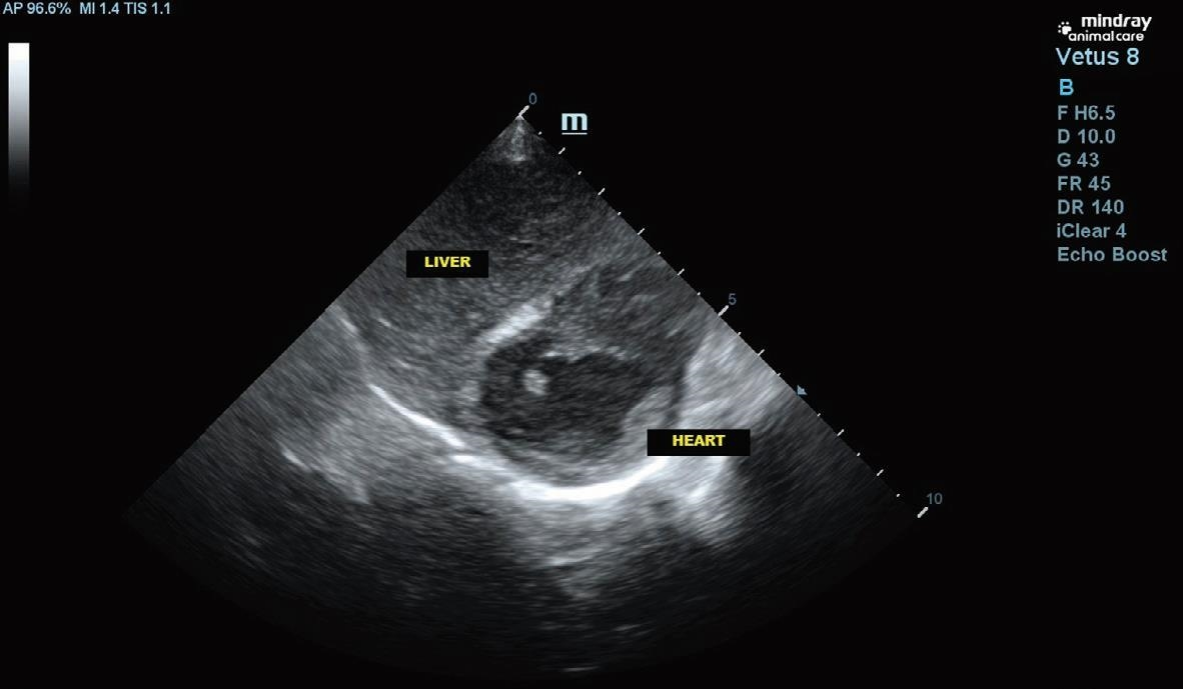
Thoracic ultrasound showing anomalous relationship between the left medial and lateral liver lobes and the pericardium.

The patient was referred to a cardiologist, who detected the absence of heart murmurs and attenuation of heart sounds in the left hemithorax. An echocardiogram was then performed, confirming that the heart was structurally normal, despite the presence of a globular silhouette on the x‐ray. Subsequently, an ultrasound was carried out and the presence of a portion of the liver within the pericardium was observed (Figure 4).

**Figure 5:**
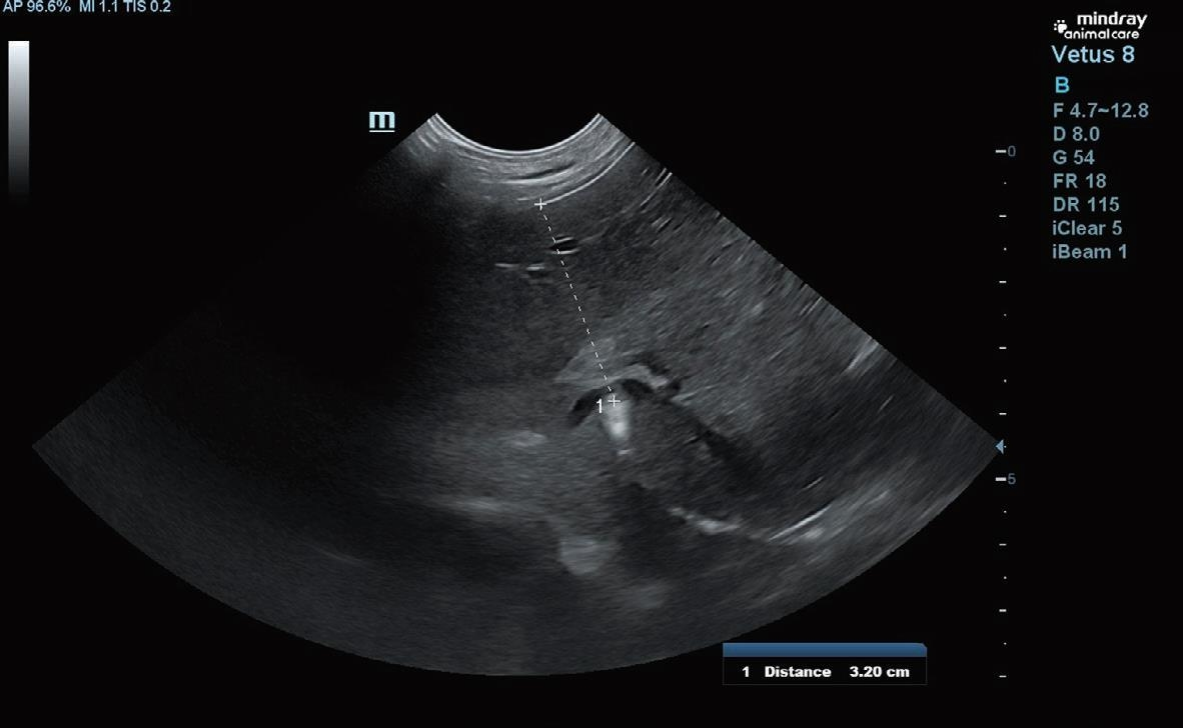
Ultrasound showing longitudinal hepatic approach in the right hypochondrium, with medial angulation of ultrasound probe.

**Figure 6:**
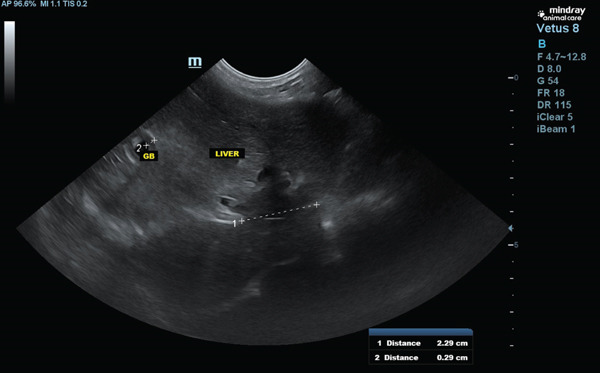
Ultrasound showing subxiphoid transverse hepatic approach, with slight cranial angulation.

A thoracoabdominal ultrasound was performed, revealing a loss of continuity in the hyperechoic line representing the diaphragm, displacement of the cranial portion of the left, medial, and lateral hepatic lobes, which were found in the thoracic cavity, affecting the caudal vena cava foramen. The dimensions of the defect measured approximately 3.20 cm × 2.29 cm, leading to a diagnosis of PPDH (Figures 4, 5, and 6).

### 2.2. Therapeutic Approach

As a treatment for PPDH, surgical intervention was considered. The drug strategy of choice consisted of premedicating the patient with medetomidine (5 *μ*g/kg) and buprenorphine (0.02 mg/kg), both intramuscularly. Fifteen minutes before surgery, cefazolin (22 mg/kg) was administered, followed by omeprazole (1 mg/kg), both intravenously (IV). Oxygenation was provided by placement of an oxygen (O_2_) rich mask for 5 min, followed by anesthetic induction with propofol (3 mg/kg IV), which was maintained with inhaled sevoflurane volatilized in O_2_ throughout the surgical procedure. To ensure adequate gas exchange, pressure‐controlled positive pressure ventilation was employed, guided by airway pressure monitoring. It is important to note that medetomidine was chosen based on its hemodynamic stability and the fact that cardiac structure and function were preserved.

**Figure 7:**
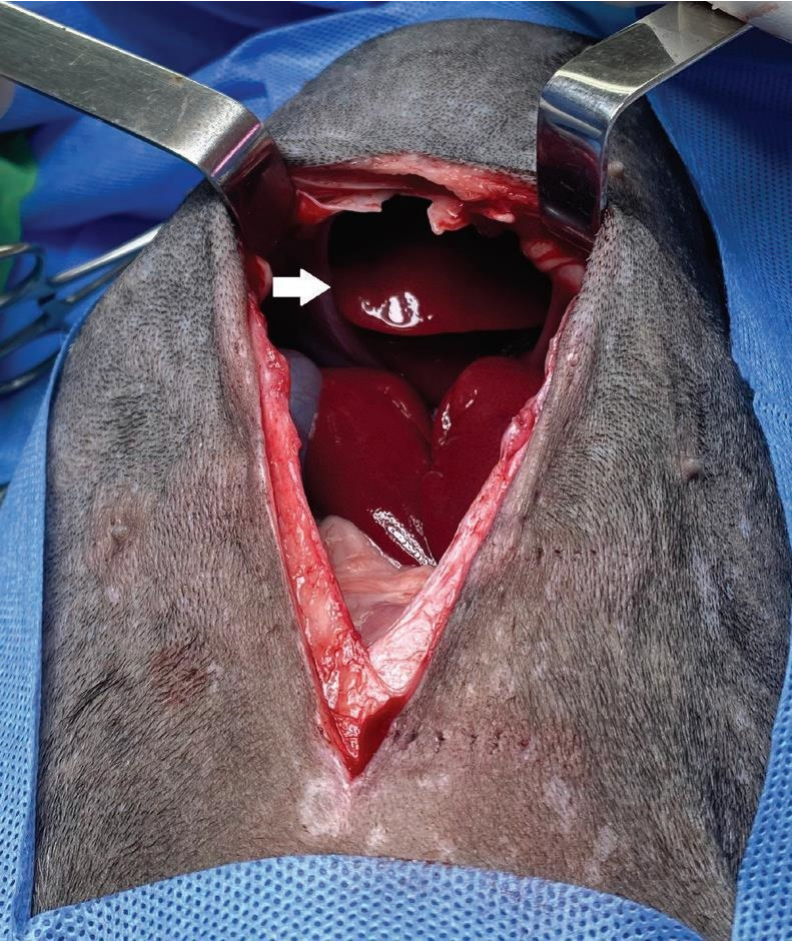
Abdominal approach and visualization of the diaphragm. The presence and displacement of the left lateral and left medial hepatic lobes are noted (arrow).

**Figure 8:**
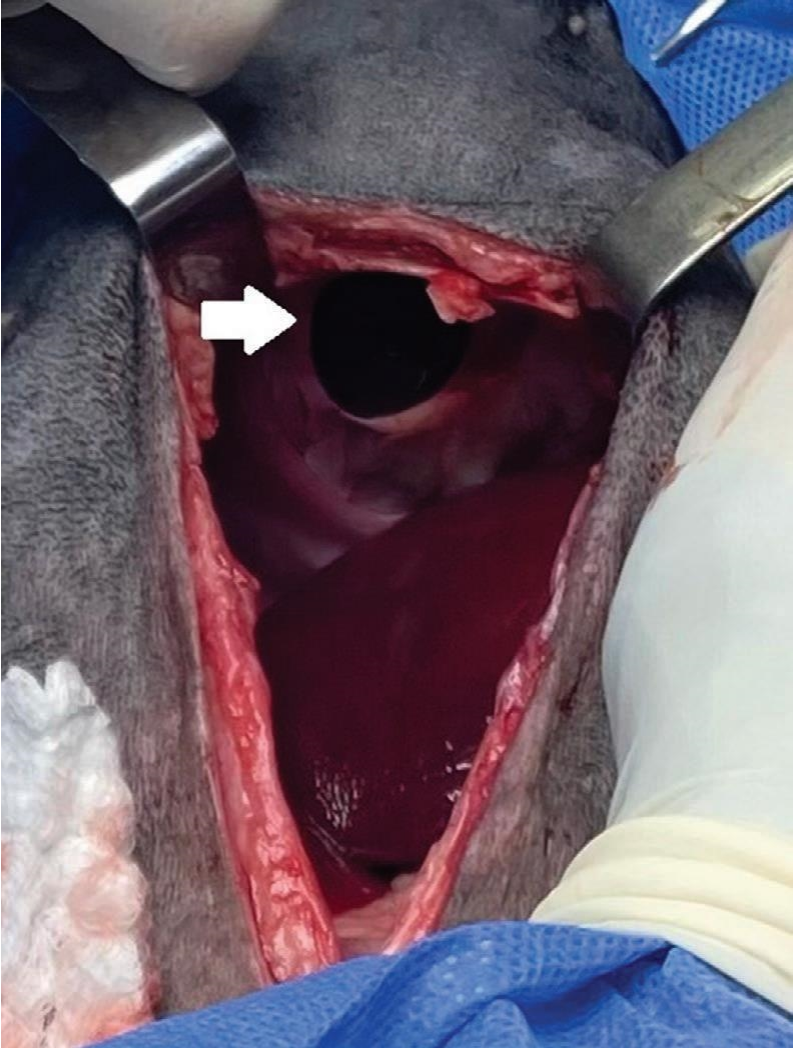
Image of the peritoneopericardial diaphragmatic hernia (arrow), once its contents have been reduced.

The patient was placed in the inverted Trendelenburg position and an incision was made in the ventral midline, cranial to the umbilicus for abdominal access. Using surgical retractors, good visualization of the diaphragm and the defect was obtained, and the displacement of the left lateral lobe and left medial lobe of the liver into the thoracic cavity could be observed (Figure 7). The contents of the hernia (the hepatic lobes) were repositioned to their normal anatomical location and protected with laparotomy compresses, moistened with saline solution (Figure 8). No adhesions were found during the reduction of the hernia.

**Figure 9:**
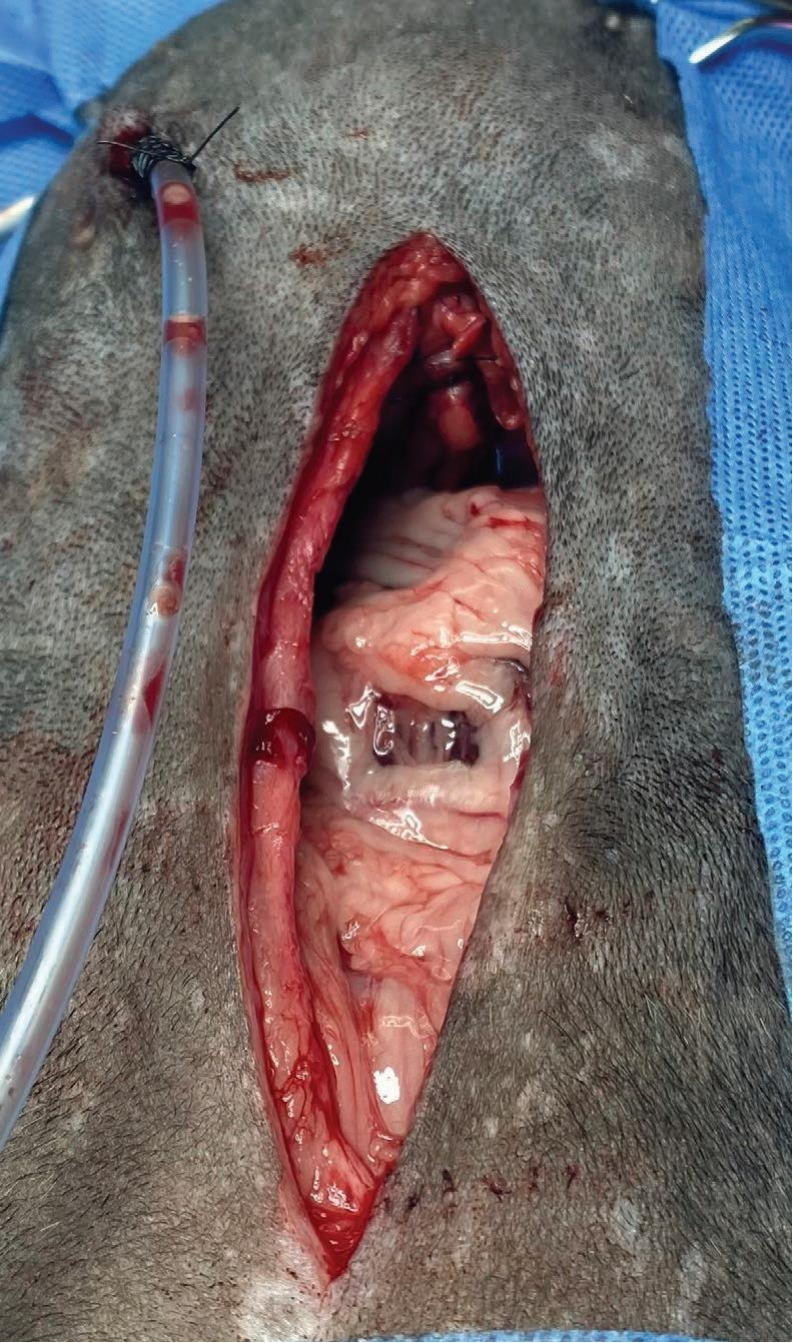
Implantation and temporary fixation of the thoracic drainage tube, by suture in a Roman sandal pattern.

**Figure 10:**
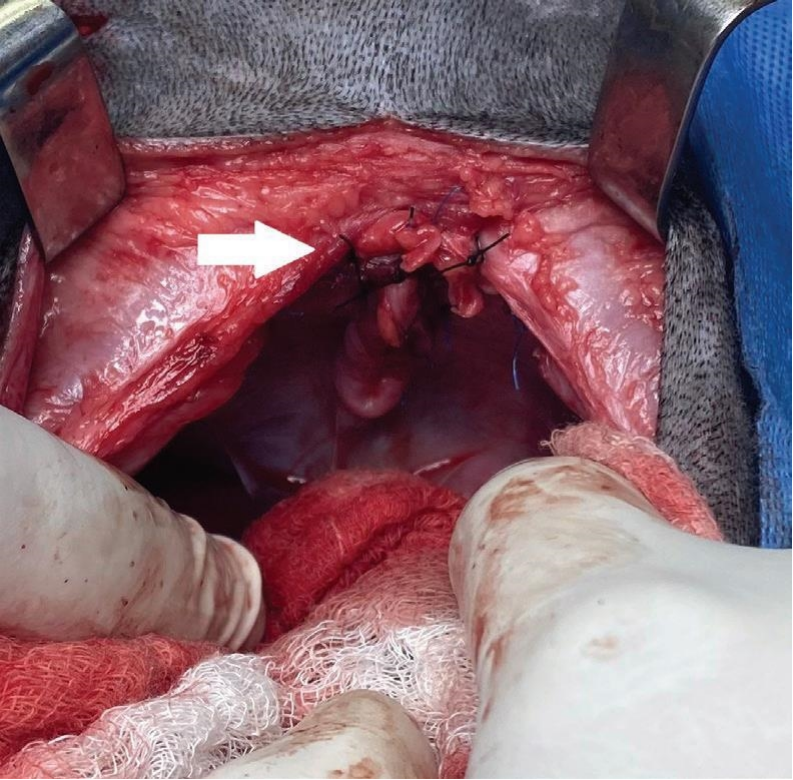
Suture pattern for peritoneopericardial hernia closure (arrow). Interrupted horizontal U‐stitches, with nonabsorbable monofilament material.

Given the size of the defect, it was decided to make a ventral advancement of the hernia, by incising the diaphragm ventrally with scissors, following the costal arch, about 3 cm on each side, to avoid excessive tension on the wound and possible postsurgical dehiscence. Prior to closure of the defect, the fluid was suctioned from the abdomen and thorax and a 12‐Fr caliber thoracic drainage tube (Thora‐Cath; Jorvet) was temporarily implanted (Figure 9). For the reconstruction, nonabsorbable monofilament suture with blunt needle (Polypropylene–Prolene; Ethicon) was used, using a pattern of interrupted horizontal U‐stitches, starting from the most dorsal portion, continuing towards the ventral and finally completing the lateral wound closure that had been previously incised (Figure 10).

**Figure 11:**
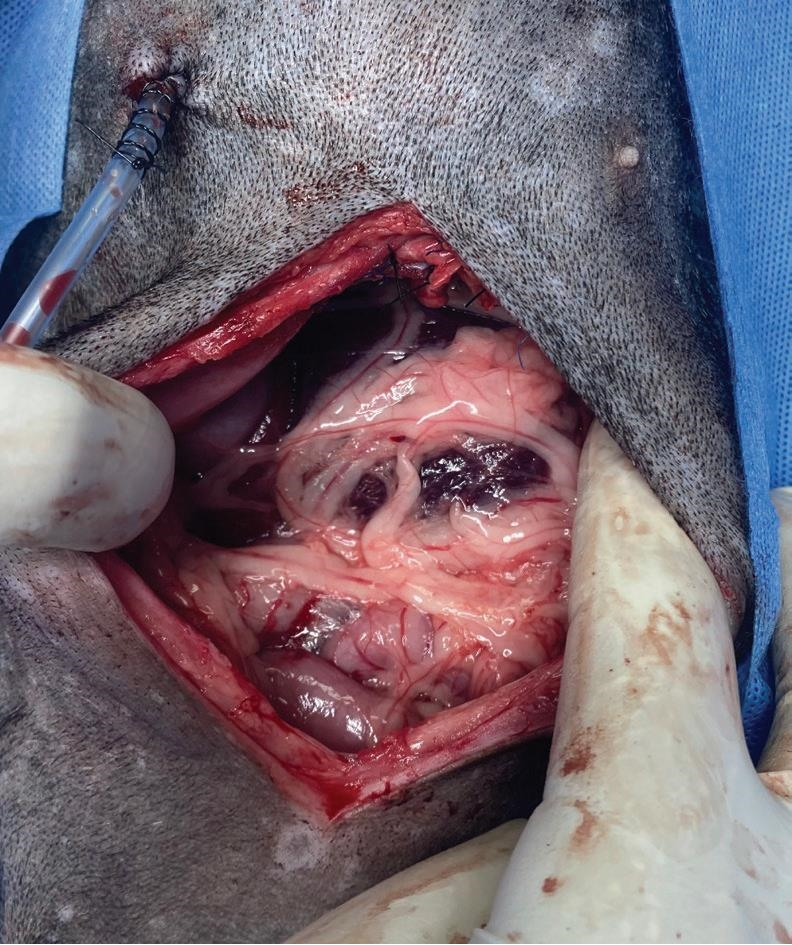
Omentum patch over the liver making contact with the diaphragm.

Before knotting the last stitch of the suture, the anesthesiologist proceeded to carry out the alveolar recruitment maneuver to avoid pulmonary collapse when closing the thorax and to allow good ventilation. An omentum patch was applied over the corrected defect (Figure 11), and abdominal closure was made using a continuous Ford′s interlocking pattern with 2–0 polyglecaprone absorbable monofilament material (Monocryl; Ethicon). Subsequently, the subcutaneous tissue was sutured with 3–0 polyglecaprone absorbable monofilament material (Monocryl; Ethicon), with a simple continuous pattern, and the skin wound closure was completed with 3–0 nylon nonabsorbable monofilament material (Nylon; Tagum), applying a simple continuous pattern. During surgical synthesis of the skin wound, metamizole (20 mg/kg) and meloxicam (0.2 mg/kg), both IV, were administered for postsurgical pain management.

### 2.3. Recovery and Follow‐Up

To monitor the patient′s evolution and check his pain scale, he was left hospitalized for the first 24 h and no complications occurred. During that stage, the patient was given omeprazole (1 mg/kg IV, q 12 h), cefazolin (22 mg/kg IV, q 12 h), and meloxicam (0.1 mg/kg IV, q 24 h). The amount of pleural fluid drained in the postoperative period was less than 5 mL, so the chest drainage tube was removed 24 h after surgery and the patient was discharged from the hospital. For home care, treatment was based on cefadroxil (25 mg/kg PO, q 12 h), meloxicam (0.1 mg/kg PO, q 24 h), and wound care with sterile saline and absolute rest, with reevaluations at 3, 7, and 14 days after surgery. The following days elapsed without complications, with improvement of the symptomatology (cough) that was the reason for the initial consultation. An ultrasound control was done 30 days after surgery, showing that the liver was in its normal anatomical position, with a slight increase in size, but with preserved echogenicity, homogeneous echo‐texture, fine edges, and smooth capsule.

## 3. Discussion

PPDH is a congenital anomaly reported in veterinary medicine, with a rare occurrence but should be considered within the differential diagnoses of young patients with symptoms of tachypnea, dyspnea, cough, anorexia, depression, vomiting, diarrhea, thinning, wheezing, dyspnea, or exercise intolerance and no history of trauma. In the case described here, the clinical presentation was mild but persistent symptoms, and it was precisely this symptomatology that prompted further evaluation. The diagnostic approach was made by combining imaging methods, such as radiographs and thoracoabdominal ultrasound, which allowed precise identification of the defect and the incarcerated content in PPDH. This is in agreement with the published studies reviewed, which state that ultrasound is a decisive tool for the diagnosis of PPDH.

The decision to use VADT was based on the size of the defect, the age of the patient, and the objective of minimizing the tension on the sutures to avoid possible postsurgical complications. In this case, this technique was particularly useful, allowing an anatomical repair without the need for muscle flaps or synthetic implants. It should be noted that no adhesions were found at the time of surgical approach, which facilitated the reduction of the hernia without complications, a situation that does not always occur in patients with this type of anomaly. Postoperative recovery was favorable, with no evidence of respiratory complications, infection, or significant accumulation of pleural fluid. The postoperative control ultrasound evaluation at 1 month showed adequate hepatic repositioning with no signs of functional alteration, confirming that the closure was effective and durable.

## 4. Conclusion

In summary, this case reinforces the opinion that VADT could be a useful and practical alternative in wide defects, especially in young patients with good tissue elasticity. Its application does not require the use of specialized instruments or complex procedures, which makes it feasible in general clinical contexts. This surgical approach is proposed as an effective option for young patients, and it is strongly recommended that similar cases continue to be documented to further validate its efficacy and assess its long‐term outcomes.

## Funding

No funding was received for this manuscript.

## Disclosure

This case report was performed as part of the employment of the author at the Institute of Experimental Surgery, Faculty of Medicine, Universidad Central de Venezuela.

## Conflicts of Interest

The author declares no conflicts of interest.

## Data Availability

The data that support the findings of this study are available on request from the corresponding author. The data are not publicly available due to privacy or ethical restrictions.
